# Impact of a Pilot Peer-Mentoring Empowerment Program on Personal Well-Being for Migrant and Refugee Women in Western Australia

**DOI:** 10.3390/ijerph19063338

**Published:** 2022-03-11

**Authors:** Shelley Gower, Zakia Jeemi, Niranjani Wickramasinghe, Paul Kebble, David Forbes, Jaya A R Dantas

**Affiliations:** 1 Curtin School of Nursing, Curtin University, Perth 6102, Australia; shelley.gower@curtin.edu.au; 2 Curtin School of Population Health, Curtin University, Perth 6102, Australia; zakia.jeemi@curtin.edu.au (Z.J.); niranji_w@yahoo.com (N.W.); davideforbes@msn.com (D.F.); 3 Faculty of Health Sciences, Curtin University, Perth 6102, Australia; paul.kebble@curtin.edu.au

**Keywords:** mentoring, refugees, migrants, women, empowerment, mental health, employability

## Abstract

The Empowerment and Peer Mentoring of Migrant and Refugee Women study (EMPOWER) examined the effectiveness of a participatory, peer mentoring program specifically tailored for migrant and refugee women to build ability, confidence, and knowledge to seek employment, a known contributor to mental health and wellbeing. Female migrant mentors (*n* = 21) supported five cohorts of mentees (*n* = 32), predominantly from Middle Eastern and Asian backgrounds, over a period of 3–12 months each between September 2019 and November 2021. The program consisted of both individual mentoring and group workshops facilitated by content experts and the research team. The mental health and wellbeing outcomes for the mentees were explored through individual interviews with both mentors and mentees. Results indicate the program helped participants develop social connections, self-esteem, self-efficacy, and personal health and safety skills. There are ongoing mental health needs in this cohort related to competing priorities and trauma. The development of trusting, respectful relationships with mentors who are committed and flexible is essential for positive wellbeing outcomes. Peer mentoring programs for migrant and refugee women can enhance mental health and wellbeing outcomes and facilitate independence. Mentors need resources to provide appropriate mental and physical health support for some groups.

## 1. Introduction

### 1.1. Global Migration Movements

The issue of global migration is significant, with over 281 million people having migrated world-wide, according to the International Migration 2020 highlights [1]. The UNHCR estimates that of this number, 33.72 million were forced to migrate due to civil conflict, internal crises, persecution, human rights violations, and climate-based disasters, with millions of civilians also facing internal displacement [1].

Australia’s humanitarian program offers resettlement to people who have been found to be refugees according to the 1951 Refugee Convention or are in need of resettlement due to evolving humanitarian situations overseas [2]. In 2019–20, the number of offshore Humanitarian Program visas was set at 18,750 for people from countries such as Iraq, Democratic Republic of Congo, Syria, Myanmar, Afghanistan, and Eritrea [3]. In 2020, the Federal Government announced it would reduce the annual refugee intake to 13,750 places as part of the COVID-19 Economic Recovery Plan [4].

### 1.2. Employment and Mental Health

Migrants’ sense of belonging to their new country is enhanced through labour force participation and is a recognised key factor in successful settlement [5,6]. For women, who may have lower access to resources, the ability to have an independent income is crucial in enabling them to become autonomous decision-makers within the family and to foster social inclusion. The lack of employment opportunities may result in isolation and loneliness [7,8]. The subsequent impact on socio-economic status can affect mental health outcomes, with a strong correlation between socio-economic status and poor mental health [9].

Resettled refugees have higher rates of unemployment than other migrant groups [10]. For refugee women in particular, employment is often temporary, or in lower-paid and lower-status roles than Australian-born women [11,12]. Skilled migrant women have lower employment rates and are less likely to be employed in positions commensurate with their qualifications than their male counterparts, or with locally born people [13], and this experience may impact negatively on their mental health.

### 1.3. Challenges Faced by Refugees and Migrants Seeking Employment

Employment policies for migrants and refugees vary between countries and visa types [14]. Employment rights for those with a skilled migrant or refugee visa are stronger than for those seeking asylum or on humanitarian visas [15]. In Australia, migrants, including temporary migrants such as those on student and work visas and refugees on humanitarian visas, have the right to work [16]. However, the difficulties faced by refugees and migrants in Australia seeking employment are well established and include language difficulties, racism, and discrimination [17], skills atrophy, and non-recognition of skills and qualifications [18,19,20]. Family commitments are highlighted both in Australia and internationally as presenting a particular barrier to workforce participation for both refugee and migrant women, including those who are highly skilled [13]. A reluctance to use formal childcare [8] as well as traditional cultural values around the carer’s role [21] limit options. A lack of networks and experience, potentially limited prior education, and employer attitudes towards cultural dress also act as barriers [10,22,23]. Poor English language competency can limit one’s ability to meet job requirements, increase social isolation and loneliness, and restrict access to further education [8,24].

### 1.4. Employment-Focussed Programs and Interventions for Unskilled Migrant Women

Previous programs that have offered social networking, work experience, and mentoring for unskilled migrants have been offered successfully in the eastern states of Australia but have not been undertaken in Western Australia and are not always specifically targeted at women [25,26,27]. Similarly, government initiatives to enhance employment outcomes for refugees do not have a specific focus on women [28].

### 1.5. Peer Support and Mentoring Programs

Peer mentoring demonstrates a belief in the value of the individual and expresses a commitment to ongoing development, capacity building, and the expectation of contributing to one’s own life through empowerment. It recognises potential, enhances growth, and encourages discovery [29]. Peer mentoring is a reciprocal process through which a more experienced individual encourages and assists a less experienced individual in developing skills, knowledge, and attitudes to be more successful. Community participation promotes a sense of belonging and improves health and well-being, while social support and peer mentoring have a protective influence on health [30].

### 1.6. Community-Based Participatory Research and Personal Empowerment

Community-based participatory research (CBPR) approaches are increasingly used to implement effective interventions through social and community action, the outcomes of which have included policy and practice changes, increased community capacity, and improvements in health inequities [30]. Wallerstein [31] defines ‘empowerment’ as “a social action process that promotes participation of people, organizations and communities towards the goals of individual and community control, political efficacy, improved quality of life and social justice” (p. 198). The ability for CBPRs to empower communities, democratise knowledge, and create social change is well-recognised [32,33]. Studies that have utilised community-based participatory approaches (CBPAs) have reported sound engagement of community stakeholders in intervention development [34].

Between 2015 and 2017, we undertook another CBPR project—The Photovoice Project—with our community partner Ishar. A recommendation from that project was the need for mentoring programs for refugee and migrant women. This project arose from that recommendation and need. The Empowering and Peer Mentoring of Migrant and Refugee Women (EMPOWER) program was co-designed with our community partner and is a participatory, peer mentoring program with refugee and migrant women to build confidence, improve knowledge to seek employment, and improve psychosocial well-being.

### 1.7. Aim

This study qualitatively examined the effectiveness of a co-designed, pilot participatory peer support intervention from the perspective of program participants. The intervention sought to have holistic impacts, such as building empowerment and improving well-being. The following objectives guided the study:

1. To develop a participatory peer mentoring program with refugee women themselves, using their existing strengths, the social capital available, and noting the systemic and structural barriers they face;
2. To identify areas where additional support is required by refugee women in employment seeking, skill development, and personal empowerment;
3. To evaluate the program and its perceived influence on refugee women’s personal empowerment, confidence, skill development, and health and well-being.

## 2. Materials and Methods

This study used a community-based participatory approach (CBPA) to develop the peer mentoring program and respond to challenges that emerged throughout the project.

### 2.1. Community Partners

In keeping with the CBPA the research team engaged with several Western Australian community organisations to help guide the project and assist with recruiting of participants. These were Ishar Multicultural Women’s Health Services (Ishar) (https://www.ishar.org.au/, accessed on 10 March 2022), Centacare Employment and Training (https://www.centacarewa.com.au/, accessed on 10 March 2022), the Indian Society of Western Australia (ISWA), the Sri Lankan Cultural Society Western Australia, United in Diversity (https://www.uidwa.org.au/, accessed on 10 March 2022) and a community contact from the Mongolian Community in WA.

### 2.2. Development of the Mentoring Program

The content and focus of the mentoring program were developed through a multi-phase process of consultation with women in the CALD community and other community representatives. A mixed methods community assessment to identify current gaps, needs, expectations, skills, and knowledge of refugee and migrant women regarding employment-seeking was undertaken with 34 women at Ishar, the results of which further informed the content and focus of the mentoring program. Secondly potential mentors provided input based on their own personal experiences in resettling and establishing connections in Australia. Lastly, representatives from local government councils provided input on maintaining sustainability.

The questionnaire and focus group data from the community assessment showed women felt they needed support with developing basic computer literacy, knowledge of legal rights and responsibilities at work, building confidence and a social network, and overcoming known barriers such as family responsibilities. Some wariness also emerged in the focus group discussions. The women reported they had been offered job preparation programs in the past that had not been fruitful.

Informal discussions with key stakeholders from the supporting community organization revealed that the disappointment expressed by participants was likely due to mismatched goals and expectations between program providers and participants regarding the provision of actual employment. Job-readiness programs do not guarantee employment and there may have been misunderstandings about this by the participants. This information highlights the importance of building a trusting and respectful relationship with the women throughout the program.

From these findings, and to maintain the flexibility to cater to individual needs, the mentoring program was culturally informed, holistic, and designed to build social capital and concepts of community participation, links to community groups and resources, emotional and social support, sense of belonging, and responsibility. In our study the needs of refugee and migrant women were different depending on their cultural background and previous educational and employment opportunities. There was deliberate flexibility in the program to allow the mentors to address and meet the varied needs of the mentees. While a suggested list of 12 topic areas was provided, mentors had the discretion and flexibility to modify the discussions and areas of focus to be of most relevance to the mentee. The anticipated outcomes were an improvement in employment skills, reduced isolation, and improvements in overall health and well-being. The final program format consisted of individual mentoring sessions approximately twice per month and group workshops covering English for employment, employment skills, and financial management. The EMPOWER program was delivered between September 2019 and November 2021, lasting between 3 and 12 months each time (Table 1). Each cohort was recruited through different community partners.

### 2.3. Participant Recruitment

#### 2.3.1. Mentors

The community partners identified potential mentors from their network of service providers who provide support to refugee and migrant women in the community. Other mentors were identified from the researchers’ personal networks and from participation in previous research projects led by the researchers. Inclusion criteria for mentors were female migrants who had established themselves in the Australian workforce and were willing to meet with a mentee approximately twice per month. There was variation in the cultural and employment backgrounds of the mentors too, and each mentor was therefore able to respond to the different needs of their mentee. A total of 21 mentors (Table 1) were recruited and trained by the research team via a 3 h training program on communication and listening skills, mentor responsibilities, problem solving and goal setting, confidentiality, and accessing external specialist counselling support for mentees who had experienced trauma.

#### 2.3.2. Mentees

Inclusion criteria were initially limited to refugee or humanitarian entrants only, but this was broadened to migrant women from non-humanitarian backgrounds with limited English and employability skills as our preliminary stakeholder meetings with Ishar and other community members identified them to be a similar group in need. Skilled women, including international students, who had been disproportionately isolated and impacted by lost employment opportunities during the COVID-19 pandemic were also included. This resulted in a non-homogenous sample with maximum variation, which enabled exploration of different cultural perspectives [35]. Using a recruitment flyer, the community partners promoted the project through their networks. All interested participants were provided with contact information for the research team to ask further questions.

An opening event was held for each cohort where mentor-mentee pairs were introduced, program resources were distributed, and clear expectations and intentions for the program were established. Mentees were invited to discuss their goals, both as a group and with their mentors. Translators were available as necessary. In keeping with CBPR, additional mentees were included in the program after it had commenced, as the need arose. A total of 32 mentees participated in the program along with 21 mentors across 5 cohorts (Table 1).

#### 2.3.3. Matching Mentors and Mentees

As much as possible, mentors and mentees were matched according to prior work, if any, or education background and area of employment interest. Initially, a deliberate choice was made to match mentors and mentees who spoke different languages to encourage English conversation. However, when some participants withdrew and others did not attend the launch events, some rearrangement of the pairings was necessitated. As a result, the decision to keep language groups separate was overturned.

### 2.4. Ethical Considerations

Ethical approval was sought and obtained from the Curtin University Human Research Ethics Committee (HRE2018-0310). All potential participants were informed at recruitment that participation was voluntary, that any relationship between them and the community partners would not be affected by their decision to participate or not participate, that they would receive a small stipend for participation, and that all personal information would be stored confidentially. Participants provided informed consent to participate in the study and for their data to be used in the research.

A code of ethics to guide conduct and behaviour between mentors and mentees was developed in consultation with community partners to facilitate a welcoming, respectful, and non-judgemental environment. Assistance was provided on an individual basis where possible to alleviate known barriers to participation such as transport [36]. Lastly, safeguards were provided for maintaining confidentiality throughout the project, including discreet use of photographs, which were only taken with consent of the participants. Written consent was obtained from mentees for participation in the program, the use of photographs, and involvement in evaluation activities such as completion of questionnaires and interviews. An orientation was provided to mentors with regard to the project, the mentoring process and the need for confidentiality and professionalism towards mentees.

### 2.5. EMPOWER Program Content

The EMPOWER pilot program was specifically tailored to provide migrant and refugee women with the ability, confidence, and knowledge to seek employment. The individual peer mentoring session topics included but were not limited to the following topics: (1) goal setting and identifying strengths, (2) Australian workplace environment, (3) interpersonal skills, self-care, and financial management, (4) legal rights and responsibilities at work, (5) interview skills, (6) developing a work search plan, (7) networking, and 8) starting your own business. Mentors and mentees were also asked to follow a set of guidelines including that mentors were not to assume a role of advocacy on their mentee’s behalf, and were not to have inappropriate expectations of mentees such as those that might be expected of an employee. At all times mentors were required to treat the mentees fairly and with sensitivity, dignity, respect, and in a non-discriminatory manner.

To supplement the individual mentoring, workshops were developed and delivered by EMPOWER staff and associated providers. Each workshop was approximately 2 h in length and was a combination of written and spoken activities, allowing for different learning styles and levels of English competency. The workshop topics were English for Employment, Employment Skills (job applications and interview skills), Financial Management and Starting a New Business (Table 1). Workshops were adjusted to suit the needs of each cohort, in keeping with the CBPR’s flexible approach, and as such content differed slightly between groups.

### 2.6. Interruptions Due to COVID-19 Public Health Measures

In February–March 2020, the COVID-19 Pandemic impacted Western Australia, with lockdowns imposed across Australia and included the public health measures of widespread closure of businesses, schools, universities, and other settings. The government mandated social distancing, online education, working from home, and social distancing [37]. With approximately one million Australians losing their jobs due to the closure of businesses during the COVID 19 pandemic, seeking employment became particularly difficult for migrant women. In addition, they were isolated and often carried the burden of home schooling children. As a result, the focus of the EMPOWER program during this period was to maintain the relationships between the mentors and mentees with an emphasis on social and emotional support, and mentors were encouraged to communicate online.

### 2.7. Data Collection and Analysis

Qualitative data were collected using mentors’ progress journals, email correspondence from mentors and mentees, and individual semi-structured interviews with mentees and mentors. The online journals were submitted regularly by mentors who responded to open-ended questions about successes, challenges and general reflections on the mentoring process and outcomes for participants. Individual interviews with 15 mentors and 10 mentees were conducted between June 2020 and January 2022. Most of the participants had attended at least 2 workshops and completed at least 8 individual mentoring sessions. As the mentoring was conducted on a needs basis not all mentees attended all available workshops. Any relevant content missed was covered in the individual sessions. Interviews were undertaken at community partner sites, or by telephone, and were audiotaped and transcribed verbatim. The triangulation achieved by using multiple data collection methods provided rich, deep information and increased the trustworthiness of the study [38].

### 2.8. Data Analysis

Thematic analysis of the qualitative data provided information on the mechanisms by which the program had influenced mental health and wellbeing. Audio recordings were transcribed verbatim. Four members of the research team used Braun and Clarke’s inductive thematic analysis technique to conduct the initial coding, undertaking continual interpretation and identification of specific themes and subthemes [38]. Within each transcript meaning units were identified and these became the initial codes. Codes were then condensed into themes. After the initial coding, continual discussion between the authors helped to refine the themes and clarify points of difference. This investigator triangulation enhanced the credibility of the findings. Initially 16 codes were identified across 8 themes and a coding framework was developed. Upon re-reading the transcripts and reading associated literature the following changes were made: ‘cultural understandings’ was condensed into the ‘social connection’ theme; ‘confidence’ and ‘identifying strengths’ were initially coded as separate themes but were combined with ‘trusting self’ to make ‘self-esteem’; occupational outcomes such as ‘paid employment’, ‘volunteering’ and ‘education’ were condensed into ‘self-efficacy’ to reflect the focus on mental wellbeing. Continual review of the coding framework over several meetings led to the finalization of themes [39].

Care was taken to ensure the analysis continued to accurately represent the views of the participants [40].

## 3. Results

Twenty-one mentors of average age 46.05 (SD = 12.26) years, residing in Australia for average 19.12 (15.97) years mentored 32 mentees of average age 41.66 (8.79) years, of which 25% have resided in Australia for 0–2 years or 3–5 years and 37.5% have resided for 10 or more years (Table 2). 25% of mentees arrived in Australia on a student visa, 21.9% on a partner visa, and 78.1% have a university degree. The most common languages spoken by mentees were Arabic (*n* = 7) and Sinhalese (*n* = 5) and for mentors, Hindi (*n* = 6) (Table 2).

Thematic analysis revealed that whilst this project aimed to enhance employability, there were also other clear positive perceived influences on the mental health and wellbeing of the mentees. Overall, participants believed the program had worked well, with positive outcomes for themselves and the mentees, even if those outcomes were not employment related.

The following four themes were identified: social connection, self-esteem, self-efficacy, and personal health and safety (Table 3). However, specific areas of poor mental health and wellbeing also emerged and highlighted areas of ongoing need.

### 3.1. Social Connection

#### 3.1.1. Reducing Isolation

For some mentor-mentee pairs, social support was deemed a greater need than employment advice. Simple social interaction outside the home was part of the support provided, and social connection became a significant outcome. In some cases, the mentorship inspired the mentee to make more of an effort to be social in her own group. In other cases, strong friendships developed within the pairs. The social connection also provided a safe space for the mentees to acknowledge and discuss their feelings.

*“I think it has resulted in [Mentee] not being isolated in her home.”*

(Mentor 3)

*“My mentor [name] has helped with me a lot in different way and never let me down, even we became friends now.”*

(Mentee 1)

*“I’m not scared to speak to new people so that’s the best thing that [Mentor] gave it to me.”*

(Mentee 8)

#### 3.1.2. Building Social Networks

For mentees with limited English skills, the mentors provided opportunities for socialising in ways that would also improve the mentee’s language skills. For mentees with particular disadvantages, the opportunity to mix with people outside their current support groups was also deemed valuable. For mentees that were more skilled and had developed their own networks, the focus was primarily on employment networks.

*“The opportunity for someone like [Mentee] to link in with people who can give her support and to have a little bit of a network of other people who are not just people with a DV background. Because I think with the current group, everyone having DV issues, you know it doesn’t, it doesn’t necessarily give her the same opportunities.”*

(Mentor 8)

*“[Mentee] was able to practice English conversation and expand her network.”*

(Mentor 3)

*“My network is now bigger than before. Now I know what the meaning of network, it helped a lot to have people around you.”*

(Mentee 2)

#### 3.1.3. Cultural Understandings

Mentees expressed interest in learning about Australian lifestyles, cultural customs, and workplace culture. Mentors were able to discuss this and suggest activities that would enhance community integration. Mentees reported that they felt cultural barriers had been lessened.

*“[Mentee] expressed interest in the events being held in Perth during the Christmas season. So, the exposure to the Australian way of life and you encourage her to experiment and enjoy that aspect.”*

(Mentor 4)

*“[Mentor]Helpful for guidance, because we don’t know most of the things, how we going with the Australian culture.”*

(Mentee 9)

### 3.2. Self-Esteem

The mentoring process enables mentees to build inner strength and self-worth. Mentees recognised their varied life experiences that enabled them during difficult situations. Exposure to a wider network built confidence. There was satisfaction with what had, for most, been an uplifting and inspiring experience. Mentors described this as a sense of ‘hope and positivity’.

#### 3.2.1. Confidence

Mentees identified that they had previously been lacking in confidence, but through mentor support had developed the confidence to engage socially and pursue their goals. Mentors described the building of confidence as being one of their initial priorities with the mentees. This, at times, requires considerable effort.

*“I was very scared and intimidated but this program just boosted my confidence basically.”*

(Mentee 3)

*“Because I was so nervous before and when I see white people I’m like oh my God, they’re just going to, you know, judge me. So, after this program I’m not scared to speak to new people so that’s the best thing.”*

(Mentee 8)

*“The most difficult part was getting that interaction going because a lot of occasions I felt that it’s the one-way street.”*

(Mentor 1)

#### 3.2.2. Identifying Strengths

Identifying mentees’ strengths was a common outcome. Often the mentees were not aware of their own strengths prior to the program and seemed surprised when they emerged through discussion.

*“[Mentee] completed the Strengths Exploration Worksheets which was a revelation to her of her many strengths.”*

(Mentor 4)

*“Our discussion has reinforced for her, how strong and self-reliant she is.”*

(Mentor 8)

#### 3.2.3. Trusting Self

The development of self-awareness and trust was identified by mentees as being useful for not only pursuing career goals but also enhancing their ability to socialise.

*“I learnt a lot about myself, I know how to discover more things in my personality that I can use it for my career.”*

(Mentee 2)

*“After Empower session I think I started to trust myself more, even I realised how other people may accept me.”*

(Mentee 1)

### 3.3. Self-Efficacy

Skills that enhanced and enabled self-efficacy and independence were also developed. A number of mentees became engaged in ventures that gave them purpose and focus, including paid employment, volunteering roles, and commencing courses of study.

#### 3.3.1. Simple Financial Management

*“I shared some resources on managing finances. We talked through just basic stuff like the Barefoot Investor was an easy book to read.”*

(Mentor 9)

*“Especially the lady was there with bank. How to save money, we do it twice I think. It was very nice.”*

(Mentee 6)

#### 3.3.2. Legal Rights Knowledge

Some mentors provided explanations of superannuation and employee rights in their discussions to help mentees working in precarious roles and foster independence.

*“It’s not just finding a job it’s helping her understand how superannuation works, what her rights are within that.”*

(Mentor 5)

#### 3.3.3. Time Management

Cultural differences in time management arose in some discussions around workplace culture. Mentors identified a need for time management skills for mentees who needed to combine family responsibilities with employment.

*“We discussed the importance of managing time not only at the workplace but also at home and in our daily life.”*

(Mentor 7)

#### 3.3.4. Occupation and Engagement

In alignment with the primary focus of the program, a number of mentees attained positions in paid employment, volunteering, or courses of study. Some mentees found positions in areas they desired, others moved to new fields or considered entrepreneurship. Mentors enabled mentees to achieve their goals by assisting with CVs and applications for work and study.

*“[Mentor] actually went through my CV and helped me around that. So that was a really good outcome so finally I was able to get a job.”*

(Mentee 7)

*“She [Mentee] even started working, it’s a small role but it’s a very good step.”*

(Mentor 2)

*“She [Mentee] has just recently joined Certificate IV in accounting and she’s well on track of you know progressing further.”*

(Mentor 3)

*“I’m studying now fashion design on TAFE, I’ll finish my certificate 4.”*

(Mentee 2)

*“I’m enrolled to do interpreting diploma.”*

(Mentee 3)

*“She [Mentor] gave me proper guidance regarding finding a job, how to make a proper CV and cover letter. And once finishing that internship… I left and found a job.”*

(Mentee 9)

### 3.4. Personal Health and Safety

The outbreak of the COVID-19 pandemic created anxiety in this cohort, particularly those with limited English skills. Mentors were able to provide clear health information in culturally appropriate ways that enabled mentees to manage lockdowns safely.

#### 3.4.1. COVID Information

*“I thought talking about COVID 19 and giving information to [Mentee] is very important. So, we spent our whole session just talking on this topic.”*

(Mentor 7)

#### 3.4.2. Cyber Safety

Mentors identified a lack of understanding of cyber safety in some mentees. By educating the mentees about the safe use of personal data online, the mentors were able to prevent harm and ensure the maintenance of privacy and dignity.

*“I told [Mentee] she should be very careful while using emails, internet or social media. She said she uses emails and Facebook a lot but she didn’t know it can be unsafe to share personal information on it.”*

(Mentor 7)

### 3.5. Ongoing Needs

#### 3.5.1. Overwhelmed with Stressors

Some mentees were managing multiple daily challenges and commitments. These included minimal financial independence, unwell extended family members, and childcare responsibilities that impeded their capacity to look for and commit to employment. Often, mentors had to assist mentees in addressing these competing priorities before moving on to the process of job seeking.

*“Minimal family support and no childcare support available.”*

(Mentor 1)

*“She had personal health problems and some financial difficulties.”*

(Mentor 11)

Complex physical and mental health issues, domestic violence situations, family law court matters, and mentees trying to help family members in danger in their home countries all contributed significantly to mental health concerns. One mentor felt conflicted by her role of helping with employment and wondered whether she should have been advocating for social services instead. Mentees also faced significant financial issues that took priority over, and sometimes precluded, seeking employment. The skills and training required to obtain employment, such as a driver’s license or completing a training course, are out of reach for some mentees due to the financial cost.

*“How do I help a mentee who cannot pay the rent?”*

(Mentor 9)

*“Difficult situation as her mother has disappeared back home and she cannot go back to look for her as her ex-husband will not sign for her children to have passports.”*

(Mentor 8)

*“I observed that she is quite depressed and isolated. She has very low self-esteem and also she has some health issues.”*

(Mentor 7)

#### 3.5.2. Desire for Mental Health Support

A number of mentees expressed a desire for more mental health support. Specific assistance was requested with managing loss, health issues, and parenting in a new culture.

*“Some people are depressing and like you know, mental, mental health part. If you guys can cover those areas, that would be great.”*

(Mentee 8)

*“I think mental health is important for us like migrant, how to raise a kid for migrant, even I think about psychology sessions. It will be very good if you provide a program.”*

(Mentee 1)

## 4. Discussion

The EMPOWER program utilized the motivation of the mentors and mentees to build confidence, connections, and skills with a focus on self-efficacy and job-readiness. Participants who completed the program reported improved social connection through reduced isolation, a greater personal and professional network, and attendance at community events that facilitated intercultural contact. Self-esteem was enhanced via improved self-confidence, the recognition of personal strengths, and greater self-awareness. Personal self-efficacy was built through improved knowledge of financial management, legal rights and responsibilities at work, and time management skills both at home and in the workplace. Several participants found positions in paid employment, volunteer roles, or commenced a course of study that enhanced their self-worth. There were several key components in the mentoring relationships that led to the following positive mental health outcomes: trust, support, flexibility, and commitment from mentors.

Mentors identified the development of trust as being critical in making the mentee feel secure, and strong personal connections were made. Trust is essential for an effective mentoring relationship [41] and may also influence mentoring behaviours [42]. Mentees who demonstrate a willingness to learn, openness, and an ability to set realistic goals are considered trustworthy by mentors who may be more willing to engage [42]. Positive emotional connections and attachments with mentees also enhance trust (Allen and Eby 2008) [43], and activities that encourage connection and understanding within pairs early in the mentoring relationship are recommended [42].

Support and encouragement were highly valued by the mentees, most of whom felt they could rely on their mentor despite difficult circumstances. The mentors’ perseverance allowed mentees to develop their skills and identify abilities and goals [41,44]. Mentors respected and validated the lived experiences of the mentees, demonstrating acceptance and confirmation [44].

The mentoring process enables mentees to build self-awareness and develop a sense of trust in themselves. In some cases, this extended to greater trust in the broader Australian community. By acknowledging mentees’ strengths, the EMPOWER program enabled mentees to confirm their inner identity and, at the same time, promoted a sense of place in Australia. This aligns with Adolfsson (2021), who studied migrants’ sense of belonging in Sweden and found that ‘multiple memberships between groups need not be contradictory but rather an expression of different spheres of inhabitance’ [45]. A sense of belonging is a key component of mental health [45].

A sense of belonging is sometimes used interchangeably with the concept of integration. The term ‘integration’ has multiple levels of application and is used differently depending on the context. It can be a contentious term with negative connotations and is sometimes proposed as a solution to community discord [46]. However, Ager and Strang posit that integration can be both a goal and a process and identify ‘indicators of integration’ as access to the social determinants of health such as housing, education, and employment, as well as social relationships and connections [47]. In this study, by building social and cultural capital, the peer mentoring process empowered women to overcome isolation and hesitancy and acknowledge pre-existing strengths that enabled greater participation in society.

As per the participatory, holistic, and inclusive design of the program, mentors tailored their support based on the individual needs, abilities, opportunities, and agency of the women, using culturally appropriate and sensitive approaches. For some mentees, this could include helping them gain recognition of tertiary qualifications. For other mentees, a much simpler level of support was required, such as helping them compile a list of known skills, reviewing CVs and providing an opportunity and avenue to discuss their hopes and challenges. Better outcomes were identified in mentees with a degree of pre-existing agency, such as holding formal qualifications, having sound English language skills, and an adequate level of self-confidence. Mentees with those assets made the most progress towards achieving employability. Agency has been shown to assist migrants with the navigation of unpredictable and precarious employment environments [48,49]. In a study of Polish migrants in the UK, Szewcayk, (2013) [49] found that graduates used their personal agency to create career trajectories for themselves in response to changeable employment markets. The use of this personal capital was necessary to manage the unpredictability of their employment journeys. Similarly, May (2019) [48] found that undocumented asylum seekers in France used agency to make the most of the limited opportunities available to them, although structural factors ultimately restricted their options. In the current study, mentors were able to build on the existing agency of the mentees with a targeted approach to help achieve specific goals. Unskilled mentees with limited language skills faced greater hurdles and presented more challenges to the mentors, who sometimes needed to work hard to overcome initial barriers and build trust. Not all mentors were successful in establishing relationships, primarily due to the considerable barriers faced by the most vulnerable mentees.

Some mentees were managing significant stressors. These include financial problems, physical and mental health issues, and difficult family situations. Mentors need to be committed and flexible to respond to mentees’ needs, which may be complex. Personal and emotional issues have been shown to hamper the mentoring process [50], and mentors need resources and support to learn new strategies and direct mentees to appropriate resources. When mentors can respond to challenges and obstacles, the mentoring relationship is more positive [41]. When mentors can understand their mentee’s worldview and social environment, they are more able to assist the mentee to develop resilience and agency. Mentors in this study sometimes struggled with the complexities of the mentees’ additional well-being and family needs outside the employment sphere. Mentors need support to refer mentees to professional services where necessary. Refugee and migrant women have been identified as experiencing higher rates of mental health problems than the mainstream Australian population. This may be a result of isolation due to childcare responsibilities, fewer opportunities for social activities, and a possible lack of language skills [16,51]. Separation from family overseas, and difficulties adapting to gender norms in the host country, are also identified as contributing to poor mental health [52].

For the more vulnerable mentees, the benefits of the program were social connection and a greater sense of belonging. The program was designed to enhance employability, but achievement of this goal is mitigated by both the personal attributes of the mentee and systemic barriers to employment, such as a lack of work experience and discrimination [10,53,54]. Not all mentees made progress towards finding employment. Program organizers and mentors must be clear in their communications about setting realistic goals and focusing on empowerment and building self-efficacy. Mentee expectations must be identified and managed where necessary to enhance the development of trust and connection.

### 4.1. Limitations and Strengths

A strength of this research was the connection that the authors had to various community organizations, allowing for access that would otherwise be difficult to attain. The community organizations assisted with recruitment and provided venues for workshops. A limitation of this study is the small sample size; therefore, the results presented are not generalizable; however, as this was a qualitative study, the depth of information, transferability, and credibility of the study were important.

Another limitation was the impact of COVID on the project, which made it difficult for mentors and mentees to meet in person. Difficulties in recruiting and retaining refugee women from different ethnic backgrounds made it necessary to expand our sampling inclusion criteria to non-refugee women. However, this is in keeping with the community-based participatory approach, which is flexible to meet the needs of participants and respond to emerging challenges. Non-refugee migrant women face similar challenges to refugee women regarding employment, and these were exacerbated by COVID-19.

### 4.2. Recommendations

Our study and the findings highlighted some recommendations for future mentoring programs. These include providing trauma-informed training to mentors and an orientation program for mentees to improve their skills in interacting and working with a mentor for mutual benefit. Mentoring programs for refugee and migrant women also need to acknowledge and work with the systemic, structural, and practical barriers to success faced by this cohort of women. Recommendations for policy include offering peer mentoring programs as a non-pharmacological mental health support and intervention for refugee and migrant women that enhances psychosocial well-being. Recommendations for research include having longitudinal studies over time to assess program effectiveness and influence on wellbeing. Future studies could evaluate the effectiveness of a work experience and internship program on employment and mental health outcomes of this cohort.

## 5. Conclusions

The EMPOWER peer mentoring program aims to enhance employability and networks for vulnerable refugee and migrant women. The pilot program provided opportunities for social connection, built mentees’ confidence and self-worth, and improved self-efficacy. Mentees with pre-existing agency had better employability outcomes. The most vulnerable mentees sometimes faced barriers to full participation in the program. Mentors with lived experience of migration were critical to providing validation and acknowledgment of the mentees’ stories. Ongoing trauma and mental health issues have caused some ongoing barriers to employment, and further resources are needed in this area. With adequate training and support, peer mentors can promote the mental health of refugee and migrant women through improvements in community participation. Despite the challenges of resettlement and integration, there remains a strong sense of community and family connection among refugee and migrant women, as well as a desire to learn new skills, gain further education, and contribute economically to their new homeland.

## Figures and Tables

**Table 1:** Table of participant cohorts and workshops delivered.

Group Duration	Participants	Workshops Delivered	Attendance
Group 1 commenced September 2019	10 mentees 9 mentors	English for Employment Employment Skills Financial Management	9 mentees 10 mentees 10 mentees
Group 2 commenced March 2020	6 mentees 5 mentors ^1^	English for Employment Employment Skills Financial Management	5 mentees 4 mentees 5 mentees
Group 3 commenced between August 2020	8 mentees 7 mentors ^1^	Financial Management and Starting a New Business ^2^ Employment Skills ^2,3^	5 mentees 7 mentees
Group 4 commenced between April–August 2021	5 mentees 1 mentor		
Group 5—group mentoring sessions held between September and October 2021	4 mentees ^4^ 2 mentors	Session One ^3^ Session Two ^3^ Session Three ^3^	4 mentees 4 mentees 3 mentees

^1^ A mentor from Groups 2 and 3 also participated in Group 1. ^2^ Groups 3 and 4 had workshops delivered at the same time. Participants in these groups also did not require an English for Employment workshop.^3^ Workshops and sessions were delivered in mixed, in-person (face-to-face) and videoconference format. ^4^ One of the mentees also participated in Group 3.

**Table 2:** Characteristics of participants.

Mentors		Mentees	
Total Mentors	21	Total Mentees	32
Country of Origin	N	Country of Origin	N
Australia ^1^; Bangladesh ^1^; Egypt ^1^; England ^1^; France ^1^; Italy ^1^; Lebanon ^1^; Mongolia ^1^; Nepal ^1^; Serbia ^1^; Singapore ^1^	11	Bangladesh ^1^; China ^1^; Ethiopia ^1^; Lebanon ^1^; Libya ^1^; Lithuania ^1^; Pakistan ^1^; Scotland ^1^; Somalia ^1^; Thailand ^1^; Togo	11
India	8	Egypt	3
Sri Lanka	2	India	2
		Iran	2
		Iraq	2
		Malaysia	2
		Mongolia	3
		Philippines	2
		Sri Lanka	5
Age in years		Age in years	
Mean (SD)	46.05 (12.26)	Mean (SD)	41.66 (8.79)
Range	26–70	Range	25–62
Years in Australia		Years in Australia	
Mean (SD)	19.12 (15.97)	0–2	8
Range	2.5–63	3–5	8
		6–9	4
		10 or more years	12
Highest Level of Education	N	Highest Level of Education	N
Technical and Further Education (TAFE)/Technical College	2	Never attended school	1
University	19	7–9 years of schooling	2
		12 or more years of schooling	2
		Trade or technical qualification beyond school	2
		University degree	25
Main Language Spoken	N	Main Language Spoken	N
Arabic	2	Amharic ^1^; Bangladeshi ^1^; Ewe ^1^; Farsi ^1^; Lithuanian ^1^; Mandarin ^1^; Persian ^1^; Scottish ^1^; Somali ^1^; Tagalog ^1^; Tamil ^1^; Thai ^1^; Urdu ^1^	13
Bangladeshi ^1^; French ^1^; Italian ^1^; Malayalam ^1^; Mandarin ^1^; Mongolian ^1^; Nepali ^1^; Serbian ^1^; Sinhalese ^1^; Tamil ^1^; Urdu ^1^	11	Arabic	7
English	2	English	3
Hindi	6	Mongolian	2
		Sinhalese	5
		Telugu	2
Industry of Employment	N	Visa Category (top 3)	N
Aged Care and Disability	1	Partner	7
Community Services	6	Refugee	4
Finance	1	Student	8
Government	1	Other	13
Hospital	2		
Self-employed/Freelance	6		
Tertiary Education	4		
		Employment status pre-program	N
		Employed	9
		Unemployed	23

^1^ Count of one i.e., *N* = 1.

**Table 3:** Perceived impact of peer mentoring program on mental health of refugee and migrant women.

Theme	Sub-Theme
Social Connection	Reducing isolation Building social networks Cultural understandings
Self-esteem	Confidence Identifying strengths Trusting self
Self-efficacy	Simple financial management Legal rights Time management Occupation and engagement
Personal health and safety	COVID-19 information Cyber safety
Ongoing needs	Overwhelmed with stressors Desire for mental health support

## Data Availability

The datasets produced and analyzed for this study are not publicly available but are available from the corresponding author on reasonable request.
